# Neglect of relevant treatment recommendations in the conduct and reporting of a laser therapy knee osteoarthritis trial: letter to the editor

**DOI:** 10.1186/s12891-020-03902-1

**Published:** 2021-01-12

**Authors:** Martin Bjørn Stausholm, Jan Magnus Bjordal

**Affiliations:** Department of Global Public Health and Primary Care, University of Bergen, Bergen, Norway

**Keywords:** Critical appraisal, Knee osteoarthritis, Laser therapy, Letter to the editor, Randomised clinical trial

## Abstract

We read with interest the article by Gomes et al. entitled: “*Exercise program combined with electrophysical modalities in subjects with knee osteoarthritis: A randomised, placebo-controlled clinical trial*”. Gomes et al. concluded that the low-level laser therapy (LLLT) did not reduce knee osteoarthritis pain when applied as an adjunct to exercise therapy. We argue that Gomes et al. neglected relevant laser treatment recommendations in the conduct and reporting of the trial.

Gomes et al. did not state the Joules per treatment spot applied. We calculated the Joules applied from other laser information in the report and found that it is too low of a dose according to the World Association for Laser Therapy (WALT) guidelines. Furthermore, we have published a meta-analysis of 22 placebo-controlled trials demonstrating a significant difference in pain-relieving effect between doses in adherence and non-adherence to the WALT guidelines. However, neither the WALT guidelines, nor our meta-analysis was mentioned by Gomes et al.

Moreover, Gomes et al. did not state whether the output power of the laser device was measured, and this is concerning because in the city of São Paulo, where the trial was conducted, most laser devices have been found to deliver less of a dose than specified by the manufacturers.

In summary, we found that the best available evidence regarding effective and ineffective LLLT dosing from systematic reviews was neglected in the conduct and reporting of the trial, and that the laser device may not have been calibrated.

We read with interest the article by Gomes et al. entitled: “*Exercise program combined with electrophysical modalities in subjects with knee osteoarthritis: A randomised, placebo-controlled clinical trial*”. Gomes et al. concluded that the addition of low-level laser therapy (LLLT) “… *did not increase the clinical benefit after 8 weeks of treatment (primary and secondary variables) when combined with an exercise protocol for knee osteoarthritis*.” [1].

We argue that the results of the trial were not interpreted in the light of what was already known in terms of LLLT dosing.

We are surprised that Gomes et al. did not state the Joules per treatment spot applied [1] since this has been found to be a crucial factor in LLLT [2–4]. However, this dose parameter can be calculated from other LLLT information in the report. Gomes et al. stated that a 904 nm wavelength laser device with a probe (spot) size of 0.1309 cm^2^ was utilized in skin contact mode and that the energy density was 6 J/cm^2^ [1]. This means that the dose per treatment spot applied was 0.78 J (6 J/cm^2^*0.1309 cm^2^ = 0.78 J).

In the World Association for Laser Therapy (WALT) dose guidelines, irradiating the osteoarthritic knee with at least 1 J of 904 nm wavelength laser per treatment spot is recommended [5]. Our research group has published a systematic review and meta-analysis with 22 placebo-controlled trials on the topic. Here, we initially found that pain was overall significantly reduced by LLLT compared to placebo-control. Subsequently, we sub-grouped the trials using the WALT recommendations for LLLT dose per treatment spot and this revealed a significant dose-response relationship; the pain-relief provided by the recommended LLLT doses was highly significantly superior to placebo even at follow-up 12 weeks post-therapy, and the difference was greater than 20 mm on the 0–100 mm visual analogue scale from the final 4–8 weeks of therapy through follow-ups 6–8 weeks post-therapy. Importantly, we found that 904 nm wavelength laser doses recommended against by WALT (lower than 1 J per treatment spot) provide no or little positive effect in knee osteoarthritis [3].

The dose applied by Gomes et al. does not satisfy the WALT recommendations and our LLLT dose-response meta-analysis can explain the negative findings. However, neither the WALT recommendations [5], nor our systematic review [3] is mentioned by Gomes et al. [1]. Gomes et al. claimed that they used a similar dose to that applied by Hegedus et al. However, Hegedus et al. stated that they applied 6 J per treatment spot, not 6 J/cm^2^ [6]. It is important to understand that J/cm^2^ is equivalent to J per treatment spot only in instances where the laser beam covers exactly 1 cm^2^, which it rarely does.

Gomes et al. did not state whether the output power of the laser device was measured. It is a major concern that in the greater São Paulo area of Brazil, where the study by Gomes et al. was conducted, 59 of 60 laser devices tested delivered less of a dose than specified by the manufacturers [7]. We conclude that is very likely that the dose used in this study is ineffective.

In summary, we found that the best available evidence regarding effective and ineffective LLLT dosing from other trials was neglected in the conclusion by Gomes et al. and that their laser device probably was not tested and most likely delivered an ineffective dose.

## Data Availability

Not applicable.

## Abbreviations

LLLT: Low-level laser therapy
WALT: World Association for Laser Therapy
